# Surgical Stillness—When, Why, and How?

**DOI:** 10.3389/fsurg.2019.00061

**Published:** 2019-10-30

**Authors:** Jacob Rosenberg, Thomas Fuchs-Buder

**Affiliations:** ^1^Department of Surgery, Herlev Hospital, University of Copenhagen, Copenhagen, Denmark; ^2^Department of Anaethesiology, Hôpitaux de Brabois, Nancy, France

**Keywords:** neuromuscular block, safety, collaboration, anesthesia, surgery, laparoscopy, robotic

## Introduction

When surgery is performed close to delicate structures, surgical stillness may be imperative. If the patient suddenly moves during a surgical procedure, whether it is body movements or just simple cuffing or sudden abdominal muscle contractions, then the surgical instrument may accidentally damage nearby structures. That is why we for certain procedures need surgical stillness.

## When

Typical areas where surgical stillness seems to be necessary are surgery in the retroperitoneum because of important structures such as the cava vein or abdominal aorta as well as numerous other delicate structures that could be injured by a sudden patient movement. This also applies for surgery close to the liver, spleen, and pancreas, such as bariatric surgery, fundoplication, gastric surgery, liver resection, splenectomy, pancreatic procedures, and many more. For robotic procedures, which are laparoscopic surgery assisted by a surgical robot, then the surgical instruments cannot be removed immediately if the patient is coughing. It is therefore routine in many countries to utilize deep neuromuscular blockade for robotic surgical procedures to prevent the patient from having any muscle contractions during the procedure (1).

Other specialities also have similar issues with sudden movements. This may include ophthalmic surgery, ENT procedures, neurosurgery, neuroradiology, ablation (heart), and advanced endoscopic gastrointestinal procedures such as mucosectomy and ERCP. The list is not final, but it is important to realize that there are numerous surgical procedures where surgical stillness is very important for patient safety.

## Why

Every surgeon has experienced injury to his or her own fingers or the fingers of the surgical assistant because of sudden patient movement during surgery. This applies for open surgery, whereas during laparoscopy there is no injury to the surgical staff but the injury to the patient can be devastating if the patient has sudden movements. Surgeons therefore consider this issue with great seriousness and worry.

Another biproduct of sudden patient movement is that the operation has to be postponed for some minutes until the patient has been relaxed. Thus, the total duration of surgery will probably be increased if there are sudden patient movements.

During laparoscopy, a sudden abdominal muscle contraction will move the laparoscopic instrument inwards with potential organ injury. The reason for this is because of the slight friction through the laparoscopic trocar, so the instrument will move together with the abdominal wall. Therefore, it is part of the routine training program for laparoscopic surgery in many countries to immediately remove all instruments when inwards the patient is coughing. We must realize, that this is a sign of inadequate anesthetic technique and the inclusion of such measures in the training program for laparoscopic surgery should be regarded as a failure.

## How

The first step to avoid sudden patient movements during a surgical procedure is to assure an appropriate level of anesthesia. Today, anaesthesiologists have a broad choice of hypnotics and analgesics with a rapid onset and short-acting profile. Moreover, devices such as a BIS monitor allow now to assess the depth of anesthesia. However, despite an appropriate level of anesthesia sudden patient movements may still occur, especially during laparoscopic surgery where neuromuscular blockade is not always standard, suggesting that hypnotics and analgesics cannot reliably compensate for neuromuscular blockade. Indeed, sudden contractions or impaired visibility despite an appropriate level of anesthesia as indicated by the BIS monitor has been shown in a randomized trial in patients undergoing laparoscopic cholecystectomy (2 x 25 patients) (2). In this trial, the investigators measured several sudden diaphragmatic contractions, sudden abdominal muscle contractions and instances with inadequate visibility at intrabdominal pressure at 15 mmHg. The total number of patients having these adverse events was 12/25 in the no neuromuscular blockade group and in one of them a perforation of the liver capsule with the trocar, leading to important bleeding, occurred. In the deep neuromuscular blockade group, adverse events were observed in 1/25 (*p* < 0.001). Another randomized trial in patients undergoing laparoscopic hysterectomy (2 x 55 patients randomized to moderate vs. deep neuromuscular blockade) found that 12/55 patients in the moderate group experienced contractions in the diaphragm or abdominal muscles compared with 0/55 in the deep neuromuscular blockade group (*p* < 0.001) (3). Similarly, the number of patients having insufflator alarms as a sign of sudden muscle contractions were 10/55 in the moderate blockade group and 0/55 in the deep blockade group (*p* = 0.001). Finally, 8/55 patients in the moderate blockade group experienced increased abdominal wall tension compared with 0/55 patients in the deep blockade group (*p* = 0.006). Thus, it seems obvious that together with an appropriate level of anesthesia deep neuromuscular blockade may be a valuable tool to inhibit sudden abdominal muscle contractions and impaired invisibility during laparoscopic surgery.

Also, of interest in this context, is the American Society of Anesthesiologists (ASA) Closed Claims Project analysis of eye injuries associated with anesthesia (4). According to their closed claims analysis, patient movements during ophthalmologic surgery resulted in eye injuries in 30% of the reported claims and more important in this context, blindness was the outcome in all of them. Most of these patients underwent general anesthesia. In many of them neuromuscular blockers were not used, and, when used, patients were not monitored with nerve stimulators. Thus, appropriate management of neuromuscular blockade is crucial to assure surgical stillness during general anesthesia and poor neuromuscular management led to poor patient outcome.

## Neuromuscular Blockade

The train-of-four (TOF) stimulation is the most widely used stimulation pattern to assess neuromuscular blockade. This mode involves four electrical impulses one after the next and the number of identifiable responses indicates the level of block. An adequate degree of neuromuscular blockade for most (peripheral) surgical procedures can be assumed until the reappearance of the second TOF response. Because of the ease of intraoperative access neuromuscular monitoring is typically applied either at the muscles of the hand (adductor pollicis muscle) or at facial muscles around the eye (orbicularis oculi muscle). However, the sensitivity to neuromuscular blocking agents varies greatly among various muscles. Thus, the dose needed to block the diaphragm and abdominal wall muscles is about 1.5 to 2 times more than the dose needed to block the adductor pollicis (5, 6). Unfortunately, the diaphragm is not accessible for non-invasive neuromuscular monitoring. Consequently, the TOF stimulation at the adductor pollicis muscle or the orbicularis oculi muscle does not allow to monitor the levels of neuromuscular block needed to avoid movements of the diaphragm or abdominal wall muscles.

To overcome this limitation of neuromuscular monitoring the post-tetanic-count (PTC) stimulation has been developed (7). It allows tactile or visual evaluation of profound neuromuscular block that does no longer respond to TOF stimulation. PTC stimulation consists of a 5 s 50 Hz tetanic stimulation followed by 15 single stimuli. PTC describes the number of detectable single stimuli, and it allows monitoring of deep neuromuscular block. The PTC response, assessed at the adductor pollicis muscle, should be less than 3/15 to exclude reliably diaphragmatic movements.

PTC was introduced in clinical practice in 1981 and thus anesthesiologists have been able to monitor deep block for almost four decades (7). However, deep levels of neuromuscular block could not be antagonized with the reversal agents (i.e., acetylcholinesterase inhibitors) available at that time because these compounds need advanced spontaneous recovery before they should be given (8). Thus, deep block was associated with an increased risk of residual paralysis and longer turnover times between two procedures. For that reason, most anesthesiologists did not apply or maintain a deep block.

Only recently a new reversal agent (sugammadex), allowing to reverse also deep block within a few minutes, was introduced in clinical practice and thus deep block can now appropriately be managed in clinical practice. Thus, it has become possible to maintain deep levels of neuromuscular block literally until the last stitch and to have minutes later a completely recovered patient ready to be extubated. Therefore, surgical stillness should no longer be wishful thinking, but it should be part of an appropriate anesthesia concept whenever it is needed. This should contribute to increased patient safety and improved surgical outcome and this underlines the effort of better collaboration and communication between surgeons and anesthesiologists. The introduction of sugammadex has opened an opportunity for a paradigm shift in modern anesthesia. However, it is not used routinely yet, probably because of a high cost compared with the cost of anesthesia drugs in general (9). With the potential to reduce complications and to decrease OR turnover time enabling a higher production the cost of this new drug may be worth it although not yet shown in clinical studies.

## Conclusion

During certain surgical procedures, surgical stillness is important for patient safety. The most logical way to ensure surgical stillness is to give deep neuromuscular blockade but until recently this was complicated by the absence of an effective and safe reversal agent. This problem has now been solved, and collaboration between surgeons and anaesthesiologists will facilitate the use of more effective anesthesia regimens where surgical stillness can be obtained when needed.

It is important to underline that the issue of surgical stillness is an issue of patient safety especially for laparoscopic surgery as well as many other surgical procedures close to delicate structures. This calls for better collaboration between surgeons and anaesthesiologists to improve patient outcome (10, 11). Other areas where a closer collaboration is beneficial include the increased involvement of anaesthesiologists in perioperative care of the surgical patient (12–15). The future lies in closer collaboration with mutual respect and knowledge across these clinical disciplines.

## Author Contributions

JR and TF-B contributed to conception and design, initial manuscript draft, critical revision, and final approval for publication.

### Conflict of Interest

The authors have previously received a speakers fee from Merck Sharp & Dohme Corp. This has nothing to do directly with the present paper.
